# Effect of SALAD Technique on CPR Quality During Intubation in Contaminated Airways: A Randomized Controlled Manikin Simulation Study

**DOI:** 10.1155/emmi/8928465

**Published:** 2025-07-23

**Authors:** Li-Wei Lin, James DuCanto, Yung-Cheng Su, Chee-Fah Chong, Chi-Chieh Huang, Shih-Wen Hung

**Affiliations:** ^1^Emergency Department, Shin Kong Wu Ho-Su Memorial Hospital, Taipei, Taiwan; ^2^School of Medicine, College of Medicine, Fu Jen Catholic University, New Taipei, Taiwan; ^3^CrazyatLAB (Critical Airway Training Laboratory), Taipei, Taiwan; ^4^Advocate Aurora Medical Group, Milwaukee, Wisconsin, USA; ^5^Department of Emergency, Ditmanson Medical Foundation Chiayi Christian Hospital, Chiayi City, Taiwan

**Keywords:** airway management, cardiopulmonary resuscitation, intermittent suction, manikin, regurgitation, suction-assisted laryngoscopy airway decontamination

## Abstract

**Background:** The management of contaminated airways potentially compromises the quality of cardiopulmonary resuscitation (CPR).

**Objectives:** This study examined the effect of suction-assisted laryngoscopy airway decontamination (SALAD) compared to intermittent suction in maintaining CPR quality during intubation in a simulated scenario of regurgitation.

**Methods:** This randomized controlled manikin simulation study employed a manikin to simulate the regurgitation of gastric contents into the oropharynx during CPR. A total of 36 emergency medical technician-paramedics participated in this study. Following a 2.5 h training on the SALAD technique, all participants were randomly assigned to use either the SALAD technique (*n* = 18) or intermittent suction (*n* = 18) during intubation on the manikin. The primary outcomes were CPR quality metrics, including chest compression rate, depth, and interruption time. The secondary outcomes were intubation success rate, intubation time, and glottic visualization during intubation.

**Results:** The SALAD group demonstrated significantly higher chest compression rates compared to the intermittent suction group, both before (115.7 vs. 110.9 bpm, *p* < 0.01) and during intubation (112.9 vs. 108.4 bpm, *p* < 0.05). The proportion of compression depths ≥ 5 cm was higher in the SALAD group than in the intermittent suction group, both at preintubation (61.6% vs. 44.4%) and intubation periods (55.6% vs. 27.8%). However, these differences were not statistically significant. No significant difference was observed between the two groups regarding compression depths and interruption times. A significant decrease of 2.8 bpm was observed in the compression rate of the SALAD group during intubation compared to the preintubation period (*p* < 0.01). In the intermittent suction group, both compression rates and depths exhibited a significant reduction during intubation (both *p* < 0.01) compared to the preintubation period. Intubation first-pass success rate and intubation time were comparable between the two groups. While the best glottic visualization prior to intubation was comparable between the groups, during intubation, the SALAD group demonstrated a significantly higher proportion of complete glottic visibility compared to the intermittent suction group (72.2% vs. 22.2%, *p* < 0.01).

**Conclusions:** The SALAD technique achieved higher chest compression rates and provided better glottic visualization compared to intermittent suction during intubation in contaminated airways.

## 1. Introduction

In patients experiencing out-of-hospital cardiac arrest (OHCA), airway contamination caused by regurgitation is a common complication, occurring in approximately one-fourth to one-third of cases [1–3]. Among OHCA patients who experience regurgitation, 67%–86% of cases occur prior to the placement of a supraglottic airway (SGA) or intubation [3]. Regurgitation in OHCA patients can lead to ventilation failure, increase the risk of pulmonary aspiration, and potentially reduce survival rate [2].

The quality of cardiopulmonary resuscitation (CPR) is the most critical factor influencing the survival of OHCA patients. However, intubation may increase interruptions in CPR [4], whereas studies have suggested that the insertion of SGAs can minimize these interruptions [5, 6]. The Pragmatic Airway Resuscitation trial further demonstrated that the use of a laryngeal tube improves 72 h survival rates compared to intubation in OHCA patients [7]. Despite these advantages, SGAs provide less protection against regurgitation during CPR compared to intubation, as shown in a human cadaver study [8]. While endotracheal intubation remains the preferred method for securing the airway, its success rate decreases in the presence of airway contamination, and there is an increased risk of esophageal intubation [9].

A rigid suction catheter is typically used for intermittent suctioning to remove airway contaminants during intubation. However, continuous chest compressions can exacerbate regurgitation, thereby necessitating compromises in CPR quality to prevent regurgitation. Our pilot study, conducted using a manikin-based simulation, indicated that intubation in a regurgitated airway during CPR has a negative impact on CPR quality metrics, diminishes the success rate of the first intubation attempt, and often requires multiple attempts [10].

The suction-assisted laryngoscopy airway decontamination (SALAD) technique was developed to address the limitations of intermittent suctioning by enabling continuous suctioning during intubation [11]. Several simulation studies and case reports have demonstrated the potential utility of the SALAD technique in scenarios where the airway contains significant amounts of secretions, vomitus, or blood [12–17]. Therefore, the present study was designed as a randomized control manikin study to investigate the effect of the SALAD technique, in scenarios involving regurgitation, in minimizing the impact on CPR quality during intubation.

## 2. Methods

### 2.1. Participants

This randomized controlled manikin simulation study was approved by our hospital (Approval no. 20220903R) and was conducted in March 2024. The study participants were emergency medical technician-paramedics (EMT-Ps) employed by fire departments across Taiwan, who were recruited on a nationwide basis. None of the participants had prior experience with the SALAD technique. All participants provided signed informed consent.

### 2.2. Simulation Setup

**Figure 1:**
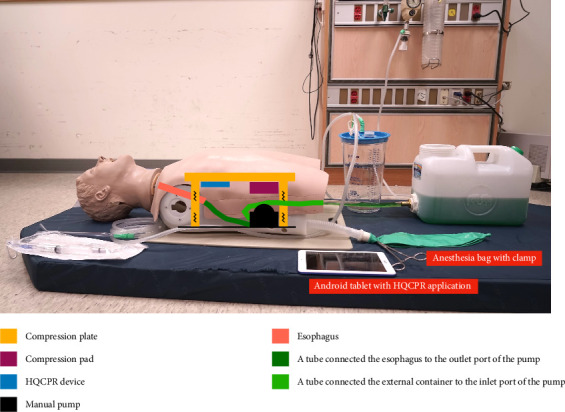
The appearance of the CPR-induced regurgitation model and its internal schematic diagram. The outlet port of the manual pump was connected to the esophagus, while the inlet port was linked to an external container via vinyl tubing. The right main bronchus was connected to an anesthesia bag through a breathing circuit, and the left main bronchus was sealed off. The compression pad and the HQCPR device were positioned beneath the compression plate. The compression pad compressed the manual pump during chest compression. The HQCPR device recorded CPR quality metrics using the HQCPR application on an Android tablet. The anesthesia bag was only unclamped for intubation checks. CPR: cardiopulmonary resuscitation.

The CPR-induced regurgitation model was adapted from an “Airway Larry” Airway Management Trainer Torso (Nasco, Fort Atkinson, WI, USA) to simulate oropharyngeal regurgitation during CPR (Figure 1). To simulate the stomach, a manual pump was affixed to the base of the manikin's torso, with a transparent vinyl tube connecting the manikin's esophagus to the pump's outlet port. A container, situated externally to the manikin, was filled with a simulated gastric content mixture, comprising 3 g of xanthan gum powder per liter of water and 1.25 mL of green food coloring gel. The aforementioned container was connected to the pump inlet port via an additional transparent vinyl tube.

A lung simulation was achieved by positioning an anesthesia bag externally to the manikin and connecting it to the right main bronchus through a breathing circuit. To prevent continuous reflux of gastric contents into the lungs during chest compressions, the left main bronchus was sealed, and the simulated lung was occluded using a surgical clamp. This clamp was only opened for the purpose of verifying the position of the endotracheal tube through the bag–valve–mask (BVM) ventilation. A compression pad was affixed to the bottom of the manikin's compression plate to compress the pump, thereby inducing reflux of gastric contents into the oropharynx during chest compressions. The thickness of the compression pad was calibrated to ensure that the compression depth would fall within the range of 0–6 cm. To simulate head movement during chest compressions, the manikin was positioned on a hospital bed mattress with a CPR board placed beneath its torso. The elastic mattress permitted the head to move during compressions, while the CPR board mitigated the compressive force absorbed by the mattress.

A high-quality CPR (HQCPR) device (Taiwan Paramedicine Service Co., Ltd., Taoyuan, Taiwan), consisting of an optical distance sensor, Bluetooth module, and battery box, was installed beneath the chest compression plate. During the performance of chest compressions, the device was programmed to measure the change in distance between the compression plate and the base of the manikin's torso. The resulting data were then transmitted via Bluetooth to an Android tablet running Android operating system Version 4.4–5.0. The HQCPR application on the Android device recorded a variety of parameters, including rate, depth, and interruptions in chest compressions.

For endotracheal intubation, a direct laryngoscope equipped with a standard Macintosh 3 blade was used. The intubation procedure involved using a lubricated 7.5 mm cuffed endotracheal tube with stylet assistance. In addition, a Yankauer suction catheter connected to a wall-mounted suction device was utilized to remove any regurgitated gastric contents from the airway.

### 2.3. Training of the SALAD Technique

All participants were provided with two and a half hours of training on the SALAD technique. The steps of the SALAD technique are as follows: First, a Yankauer suction catheter is inserted into the mouth from the right side while simultaneously lifting the jaw, controlling the tongue, and suctioning regurgitated contents from the oral cavity to the pharynx. Subsequently, the laryngoscope is then inserted, and continuous suction is maintained with the Yankauer catheter. After properly positioning the laryngoscope blade in the vallecula to expose the glottis, the Yankauer suction catheter is withdrawn and reinserted from the left side of the laryngoscope into the hypopharynx allowing for the continued suctioning of contents regurgitated from the esophagus. Finally, the endotracheal tube is correctly inserted into the trachea through the right side of the laryngoscope.

During the 2.5-hour training session, each participant performed endotracheal intubation using a C-MAC® S video laryngoscope (Karl Storz GmbH and Co. KG, Tuttlingen, Germany) with a size three Macintosh single-use blade on the Life/form® SALAD Simulator (Nasco, Fort Atkinson, WI, USA), which features the same head as the “Airway Larry” Airway Management Trainer Torso.

The initial two training rounds employed the SALAD technique with the video laryngoscope, while the third round utilized the C-MAC®S video laryngoscope as a direct laryngoscope for practice purposes. During the initial three rounds, participants were permitted to adjust their technique while being monitored via the video system. The fourth round involved intubation using a direct laryngoscope with a size 3 Macintosh blade on the SALAD simulator. The final round involved intubation on the CPR-induced regurgitation model, using a direct laryngoscope and employing the SALAD technique during chest compressions.

### 2.4. Simulation Procedure

The simulation and evaluation process was conducted immediately after the completion of the 2.5 h training. All participants were randomly assigned to one of two groups: the SALAD group and the intermittent suction group. Depending on the group assignment, either the SALAD technique or the intermittent suction technique was employed to assist in airway decontamination. A randomization process was conducted using the simple randomization method with a random number table. Those assigned to the same suction technique group constituted a resuscitation team, with each member assuming the roles of airway manager, first chest compressor, and second chest compressor in turn during three simulation sessions.

In each simulation session, the airway manager was tasked with the responsibility of administering both BVM ventilation and intubation. The first and second chest compressors performed chest compressions alternately, switching every five CPR cycles, in accordance with a compression-to-ventilation ratio of 30:2. After the initial 30 chest compressions, two ventilations were administered, with no suction performed during this phase, regardless of the occurrence of regurgitation. This approach was designed to simulate a scenario where the oral cavity was filled with regurgitant materials during intubation, thereby ensuring consistency across participants. Following the first two ventilations, intubation could be initiated while the team continued with chest compressions. The airway manager had the option to request a temporary reduction in the intensity of chest compressions, a brief cessation, or even a lightening of the compressions to facilitate intubation. Alternatively, the compressions could be continued for up to 60 cycles without interruption if necessary. In the event of an unsuccessful intubation attempt, the airway manager was obliged to administer two BVM ventilations before attempting intubation once more.

After intubation, the airway manager assessed lung expansion using the BVM to verify successful intubation. A failed intubation was defined as any esophageal intubation or three unsuccessful intubation attempts. Each simulation concluded with either successful or failed intubation. The video illustrating the simulation process for the SALAD group is provided in the Supporting Information (available here).

### 2.5. Evaluation

The primary outcomes were CPR quality metrics, including chest compression rate, chest compression depth, and interruption time. The secondary outcomes were the intubation first-pass success rate, intubation time, and glottic visualization during intubation.

Compression rates and depths were assessed during both the preintubation and intubation periods. The time of interruptions for ventilation or airway management between the two periods was also recorded. An intubation attempt was defined as the insertion of the laryngoscope blade into the mouth and its subsequent withdrawal in the event of an unsuccessful attempt, or alternatively, as the insertion of the laryngoscope blade followed by confirmation from the airway manager that the tube had been successfully inserted. Intubation time was defined as the interval between the start and the end of an intubation attempt. An interruption was defined as any cessation in chest compressions lasting more than one second.

The visualization of the glottis during CPR was evaluated using the Cormack and Lehane classification: Grade 1, the majority of the glottis is visible; Grade 2, at best, nearly half of the glottis is visible, or at worst, only the posterior tip of the arytenoids is observed; Grade 3, only the epiglottis is visible; and Grade 4, no laryngeal structures are visible [18]. The extent to which the glottis was obscured during intubation was categorized as follows: Grade 1 (0%), complete visibility of the glottis; Grade 2 (0%–49%), more than half of the glottis remains visible; Grade 3 (50%–99%), more than half of the glottis is obscured; and Grade 4 (100%), complete obstruction of the glottis.

Two video cameras were utilized to record the entire simulation process. Two independent observers reviewed the video recordings to identify intubation attempts, and any discrepancies were resolved through mutual consensus. The HQCPR application on an Android device was used to record the depths, rates, and interruptions of chest compression. Subsequent analyses were conducted using data from both the video recordings and the HQCPR application.

### 2.6. Sample Size Determination

The objective was to investigate the impact of two distinct suction techniques on the quality of CPR in scenarios involving airway regurgitation. Given the scarcity of prior research on this specific subject, we used preliminary data to estimate the required sample size. Our previous study revealed that the median chest compression rate during intubation under regurgitation conditions was 102 beats per minute (bpm). Based on the assumption of a mean compression rate difference of 5 bpm between the suction techniques, with a standard deviation (SD) of 5 bpm, the required sample size was calculated to be 17 participants per group in order to achieve a significance level of 0.05 and a power of 0.8. Given that the smallest unit of measurement in this study design is three participants per resuscitation team, the sample size was increased to 18 participants per group, resulting in a total of 36 participants.

### 2.7. Statistical Analysis

Categorical variables were presented as counts and percentages, while continuous variables were presented as means and SDs. Comparisons between the SALAD and intermittent suction groups were performed using the two-sample *t*-test for continuous variables and either the Fisher's exact test or the chi-squared test for categorical variables. Compression rate, compression depth, and intubation time were summarized as means with 95% confidence intervals (CIs). Within-group comparison of CPR quality metrics before and during the intubation period was analyzed using the paired *t*-test for both the SALAD and intermittent suction groups. A two-tailed *p* value of less than 0.05 indicated statistical significance. All data analyses and sample size calculations were conducted using MedCalc Statistical Software, Version 22.023 (MedCalc Software, Ostend, Belgium).

## 3. Result

### 3.1. Participant Characteristics

A total of 36 EMT-Ps were included in this study, with 18 EMT-Ps in each group. There were no statistically significant differences between the SALAD and intermittent suction groups regarding sex, years of EMT experience, or experience of CPR and intubation in OHCA patients (Table 1). However, a significant age difference was identified between the two groups, with the intermittent suction group being older than the SALAD group (39.6 vs. 34.3 years, *p* < 0.01).

### 3.2. Primary Outcomes: CPR Quality

Prior to intubation, the SALAD group exhibited a significantly higher chest compression rate compared to the intermittent suction group (115.7 vs. 110.9 bpm, *p* < 0.01), with an average difference of 4.8 compressions per minute. However, there was no statistically significant difference in compression depth between the two groups with both averaging 5 cm. The SALAD group exhibited a higher proportion of compressions with a depth ≥ 5 cm (61.6% vs. 44.4%), although this difference did not reach statistical significance (Table 2).

During the first intubation attempt, the SALAD group continued to outperform the intermittent suction group in chest compression rate (112.9 vs. 108.4 bpm, *p* < 0.05), with an average difference of 4.5 compressions per minute. No statistically significant differences were found between the groups regarding interruption time (2.3 vs. 2.5 s) or compression depth (5.0 vs. 4.9 cm). However, the SALAD group had a higher proportion of compressions with a depth ≥ 5 cm (55.6% vs. 27.8%), although this difference was not statistically significant (Table 2).

Regarding the participants with subthreshold compressions (< 5 cm), the mean depth of sub-5 cm compressions was 4.6 cm (95% CI: 4.4–4.8) in the SALAD group and 4.7 cm (95% CI: 4.6–4.9) in the intermittent suction group (*p*=0.197) during the preintubation period. During intubation, the mean depth was 4.5 cm (95% CI: 4.3–4.7) in the SALAD group and 4.7 cm (95% CI: 4.6–4.8) in the intermittent suction group (*p*=0.069).

A comparison of CPR quality between the preintubation and intubation periods within the SALAD group revealed a statistically significant decrease in chest compression rate by 2.8 bpm (*p* < 0.01). In contrast, the intermittent suction group experienced significant reductions in both chest compression rate (−2.6 bpm, *p* < 0.01) and compression depth (−0.2 cm, *p* < 0.01) during intubation compared to the preintubation period (Table 3).

### 3.3. Secondary Outcomes: Intubation First-Pass Success Rate, Intubation Time, and Glottic Visualization During Intubation

The first-pass success rates for intubation were comparable between the SALAD and intermittent suction groups, with both groups demonstrating a first-pass success rate of 77.8%. In addition, the mean intubation time for both groups was similar, at 16.1 and 15.9 s, respectively. In the intermittent suction group, all four cases of initial intubation failure were due to esophageal intubation (22.2%), thereby precluding any subsequent intubation attempts. In contrast, the SALAD group recorded a single case of esophageal intubation (5.6%), although this difference was not statistically significant between the groups.

Among the four participants in the SALAD group who did not achieve first-pass intubation success, three were able to complete the successful intubations on their second attempt, while one failed due to esophageal intubation. For these three participants, variations in CPR quality were observed between the first and second intubation attempts, including changes in interruption time (7.2 vs. 2.6 s), chest compression rate (116 vs. 114 bpm), and compression depth (median 5.3 vs. 5.3 cm). However, statistical analysis was not feasible due to the limited sample size.

As presented in Table 2, the best glottic visualization during CPR was comparable between the SALAD and intermittent suction groups (*p*=0.59). However, during intubation, a significant difference was observed in the degree of glottic obstruction between the two groups. The proportion of complete visibility of the glottis (Grade 1) was significantly higher in the SALAD group compared to the intermittent suction group (72.2% vs. 22.2%, *p* < 0.01). In the intermittent suction group, 11 (61.1%) subjects reported obstruction of more than half of the glottis during intubation (Grades 3–4), and two failed intubations were observed.

## 4. Discussion

This study yielded a crucial finding: In the context of a regurgitated airway, the SALAD group consistently exhibited a higher compression rate compared to the intermittent suction group, both before and during intubation. According to the 2015 American Heart Association guidelines, HQCPR necessitates the maintenance of a chest compression fraction (CCF) exceeding 60%, with a compression rate of 100–120/min, a compression depth of 5–6 cm, and a compression pause duration of less than 10 s [19]. While the SALAD group maintained a statistically higher chest compression rate than the intermittent suction group, both groups remained within the guideline-recommended range, limiting the clinical relevance of this finding. More importantly, the SALAD group demonstrated a consistently higher proportion of compression depth ≥ 5 cm, even though the difference did not reach statistical significance. This trend may reflect better preservation of CPR quality when using the SALAD technique under conditions simulating airway contamination. Notably, the SALAD group achieved significantly better glottic visualization during intubation compared to the intermittent suction group. The improved glottic visualization observed in the SALAD group suggested that this technique may help reduce cognitive burden during airway management by simplifying intubation in the presence of regurgitated material. This potential reduction in complexity and mental load could be even more impactful in the unpredictable and high-pressure setting of real-world prehospital cardiac arrests, where even small performance improvements may translate into meaningful clinical benefits.

Despite the absence of statistically significant differences in chest compression depth between the SALAD and intermittent suction groups, the mean chest compression depth in the SALAD group before and during intubation remained at the guideline-recommended level of 5 cm. This indicates that participants in the SALAD group displayed greater confidence and proficiency in managing a regurgitated airway, maintaining an optimal chest compression rate from the onset of CPR. However, the presence of regurgitated contents during intubation still resulted in a significant reduction in chest compression rate by 2.8 bpm compared to the preintubation period in the SALAD group. In contrast, the intermittent suction group experienced significant declines in both chest compression rate (by 2.6 bpm) and depth (by 0.2 cm) during intubation compared to the preintubation levels. While the SALAD technique is not without its limitations, it has been demonstrated to have the potential to mitigate the impacts of regurgitation on CPR quality in comparison to the intermittent suction technique.

The SALAD technique was observed to improve the rate of chest compressions, yet not the depth, in a manner analogous to the findings of the ENFONCE study [20]. The ENFONCE study, which included CPR-trained public and first responders, demonstrated that higher compression rates on a manikin resulted in shallower depths with a median compression rate of 114 bpm and a median compression depth of 4.5 cm. Only 46% of the chest compression rates and 30% of the depths observed were in accordance with the recommended guidelines. A study by McAlister et al. examined the performance of firefighters performing CPR in OHCA patients and revealed that the average chest compression depth was only 4.8 cm, with just 33.6% of compressions meeting the guideline-recommended depth of 5–6 cm [21]. Stiell et al. investigated that the optimal depth interval for maximum survival among OHCA patients and discovered that it ranged from 40.3 to 55.3 mm, with the peak survival rate occurring at a depth of 45.6 mm [22].

A more detailed analysis of chest compression depth in our study revealed that, prior to intubation, the proportion of compressions with a depth equal to or exceeding 5 cm was higher in the SALAD group compared to the intermittent suction group (61.1% vs. 44.4%). During intubation, this discrepancy increased further, reaching 55.6% vs. 27.8%. Although these discrepancies did not reach statistical significance, they indicated a trend toward better adherence to guideline-recommended compression depth in the SALAD group. Notably, compressions that fell below the 5 cm threshold were generally close to the target depth in both groups, suggesting that the observed differences may still have clinical relevance despite not reaching significance. The higher proportion of adequate-depth compressions in the SALAD group highlighted its potential to support higher-quality CPR during airway management in contaminated airways.

Previous studies have linked poor glottic visualization with intubation failure and multiple attempts. However, enhanced visualization in our study did not directly translate into improved CPR quality. Several factors may explain this. First, the study used manikin simulations, where participants had memorized the glottic position, facilitating intubation regardless of visualization quality. Second, the experienced and skilled EMT-Ps may have reduced the impact of the SALAD technique. This was evident as the proportion of cases with poor glottic visualization was higher in the control group (61%) than the SALAD group (5.6%), yet intubation success under poor visualization remained high at 82%. CPR performance, including compression rate, depth, and interruption time, met guideline recommendations in the control group [19]. In addition, Pilbery et al. employed intermittent suction followed by SALAD training, demonstrating reduction in intubation time with the SALAD technique [13]. In contrast, Ko et al. employed SALAD training first, then tested both techniques, and found no significant difference in intubation time [14]. This suggests that SALAD training may also enhance intermittent suction skills in managing airway contamination. Better glottic visualization may facilitate easier intubation, especially for less experienced providers, and could potentially reduce the learning curve in managing contaminated airways. While the observed improvements in CPR quality with the SALAD technique may seem counterintuitive, the findings may reflect more effective suctioning and reduced procedural interruptions rather than a direct enhancement of CPR performance. Further research is needed to explore the clinical implications of these findings, particularly in real-world settings.

In the present study, the intermittent suction group was composed of participants who were on average, older than those in the SALAD group. Prior research by Vittinghus et al. has indicated that EMTs aged 50 years and above tend to perform chest compressions at shallower depths and higher frequencies than their counterparts aged 36–50 years [23]. However, given that the average age of participants in our study groups was below 50 years, it is unlikely that age significantly influenced the quality of CPR performance.

This study did not identify any significant differences in first-pass success rates or intubation times between the two groups. These results may be explained by a number of factors. First, the SALAD group exhibited a higher chest compression rate, which could lead to a greater volume of regurgitated material, potentially compromising the efficacy of the SALAD technique. Second, the SALAD technique involved the oral cavity being occupied by a laryngoscope, a Yankauer suction catheter, and an endotracheal tube, which restricted the visual field required for the accurate placement of the endotracheal tube. Third, the studies by Pilbery et al. initially employed intermittent suction before proceeding to train the SALAD technique and subsequently performing SALAD intubation. The findings indicated that the SALAD technique resulted in shorter intubation time compared to pretraining intermittent suction [13]. However, in contrast to the results reported by Pilbery et al., the study by Ko et al., which involved training first in SALAD techniques before comparing traditional suction and SALAD techniques, showed no difference in intubation time [14]. This suggests that training in the SALAD technique also enhances intermittent suction skills in managing airway contamination.

In our preceding pilot study, we investigated the CPR quality in the context of regurgitated airways. The findings indicated that the intermittent suction technique resulted in a first-pass intubation success rate of only 35.2%. In addition, a median chest compression rate of 102 bpm and a compression depth of 5.2 cm were observed. Subsequent intubation attempts necessitated prolonged interruptions [10]. However, in the current study, the intermittent suction group increased their chest compression rate to an average of 110.9 bpm with a depth of 5 cm, achieving a first-pass success rate of 77.8%. This notable improvement in chest compression rate and first-pass success can be attributed to two primary factors: first, the participants in this study received 2.5 h of SALAD training prior to the simulation. It is postulated that the SALAD training may facilitate the performance of intermittent suction for airway contaminants. Second, continuous chest compressions were performed for 60 cycles, thus eliminating the necessity for interruptions due to the standard 30:2 compression-to-ventilation ratio. In a separate study simulating vomiting in nonchest-compression scenarios, the median intubation time was 37.1 s with intermittent suction and 26.9 s with the SALAD technique [12]. According to the guidelines, the chest compression rate should be between 100 and 120 beats per minute, with a recommended range of 15–18 s for 30 compressions. This interval is insufficient for successful intubation in a contaminated airway, particularly in a CPR context where the manikin's head is expected to move. The outcomes of the two studies may diverge considerately if the 60 compressions are uninterrupted.

The extent literature reveals a paucity of data concerning the combined effects of regurgitation on airway management and CPR quality. The majority of research has focused on the effect of intubation on CPR quality or the impact of airway contamination on the intubation success rates. Prior studies have demonstrated that intubation can interrupt CPR in OHCA patients [4], prompting numerous investigations into the use of SGAs to reduce CPR interruptions and improve outcomes in OHCA patients [5, 6]. The AIRWAYS-2 study demonstrated that there were no significant differences in the rates of regurgitation and aspiration, as well as in functional outcomes, between the i-gel group and the intubation group in OHCA scenarios [3]. In addition, the Pragmatic Airway Resuscitation Trial investigated the use of laryngeal tubes versus intubation, demonstrating improved 72 h survival in OHCA patients without differences in rates of aspiration pneumonia [7]. The available evidence suggests that regurgitation may have a detrimental impact on the survival of OHCA patients. The study by Simons et al. observed that the occurrence of emesis was associated with a reduced likelihood of survival to hospital discharge [2]. In light of the limited efficacy of SGAs in the presence of regurgitation [8], intubation remains the preferred approach [24]. Conversely, numerous studies have indicated that intubation does not necessarily result in a reduction in the CPR quality compared to the use of SGAs. The secondary analysis of the PARAMEDIC-2 trial revealed no significant difference in CCF or compression rate between intubation and the use of SGAs [25]. Pediatric CPR studies have reported that the first-pass success rate for intubation with CPR interruptions was 63%, compared to 41% without interruptions. However, this finding was not statistically significant [26]. Robinson et al. observed that in adult OHCA cases, continuous CPR resulted in a higher first-pass success rate for intubation (87% vs. 65%) compared to interrupted CPR [27]. Nevertheless, regurgitation during CPR affects more than just intubation. Our pilot study demonstrated that regurgitation could lead to decreased CCF and reduced chest compression depth. Furthermore, multiple intubation attempts prolonged the interruption times [10]. Murphy et al. reported that OHCA patients who required fewer intubation attempts exhibited better neurological outcomes [28]. In this study, the SALAD technique demonstrated its capacity to reduce the incidence of esophageal intubation and the necessity for repeated intubation attempts in the presence of airway contaminants.

The findings of this study have important implications for emergency medical practice, particularly in scenarios involving airway contamination during CPR. The use of the SALAD technique was shown to maintain a higher chest compression rate during intubation compared to intermittent suction, thereby potentially improving CPR quality in contaminated airways. Given that HQCPR is critical for the survival of OHCA patients, incorporating the SALAD technique into airway management protocols could enhance patient outcomes, particularly in cases where regurgitation is likely. Training emergency medical personnel in the SALAD technique could reduce the negative impact of airway contamination on CPR quality, minimizing interruptions and maintaining optimal compression rates, which are key factors in improving survival rates. Therefore, integrating the SALAD technique into standard practice during CPR, especially in high-risk cases, could be a valuable addition to emergency airway management strategies.

It is important to acknowledge that the study is subject to a number of limitations. First, the CPR-induced regurgitation model was developed with the objective of providing a controlled setting for research. However, this configuration is unable to reproduce the complexity and variability observed in real clinical environments. The use of a manikin does not fully replicate the intricacies of human anatomy, and the chest compression–induced regurgitation may not accurately reflect the dynamics of regurgitation in OHCA patients. The regurgitation contents also do not accurately reflect the composition of real gastric contents. In addition, allowing continuous chest compressions for 60 cycles without interruption resulted in a notable alteration in the intubation success rate for intermittent suction when compared to the results of our pilot study. This finding suggests that manikin-based simulations may not fully reflect the unpredictability of human responses during cardiac arrest situations. Second, despite the study being designed as a randomized controlled study to minimize bias and to allow for valid inferences, the relatively small sample size may reduce the statistical power of the study and increase the risk of Type II errors, which could result in the failure to detect actual effects. Third, the study's design required the termination of the simulation in the event of esophageal intubation. This resulted in four cases in the intermittent suction group, in which participants were unable to attempt a second intubation, thereby limiting the scope of investigation into the impact of repeated intubation attempts. Finally, although all participants underwent 2.5 h of SALAD training, the study did not evaluate whether this training resulted in consistent skill levels and confidence across the groups, which could potentially affect the study outcomes. Furthermore, the participants engaged in intubation practice on the same airway trainer head on multiple occasions, which may have resulted in the attainment of success even in suboptimal conditions.

## 5. Conclusion

In this randomized controlled manikin simulation study, the SALAD technique was observed to maintain CPR performance during intubation in contaminated airways, with trends toward improved compression depth and significantly better glottic visualization. While compression rate differences were statistically significant, both techniques fell within acceptable guideline parameters. The SALAD technique may reduce cognitive load during airway management, potentially improving performance under real-world emergency conditions. Further investigation is required to ascertain the impact of varying suction techniques on CPR quality during intubation.

## Figures and Tables

**Table 1:** Participant's characteristics.

Variables	SALAD group (*n* = 18)	Intermittent suction group (*n* = 18)	*p* value
Sex			1.00
Female	2 (11.1)	3 (16.7)	
Male	16 (88.9)	15 (83.3)	
Age (years)^∗^	34.3 (3.5)	39.5 (6.1)	< 0.01
EMT experience (years)^∗^	10.8 (4.4)	13.8 (7.6)	0.15
Experience of intubation in OHCA patients			0.62
< 10	11 (61.1)	8 (44.4)	
11–20	5 (27.8)	6 (33.3)	
21–50	2 (11.1)	3 (16.7)	
51–100	0 (0.0)	1 (5.6)	
Experience of intubation success rate in OHCA patients			0.42
< 50%	6 (33.3)	6 (33.3)	
51%–70%	6 (33.3)	3 (16.7)	
71%–90%	3 (16.7)	7 (38.9)	
90%–100%	3 (16.7)	2 (11.1)	
Annual experience of CPR in OHCA patients			0.89
< 5	3 (16.7)	4 (22.2)	
6–10	5 (27.8)	4 (22.2)	
11–20	8 (44.4)	9 (50.0)	
> 20	2 (11.1)	1 (5.6)	

*Note:* Data were presented as count (percentage) or mean (standard deviation)^∗^.

Abbreviations: CPR, cardiopulmonary resuscitation; EMT, emergency medical technician; OHCA, out-hospital cardiac arrest; SALAD, suction-assisted laryngoscopy airway decontamination.

**Table 2:** Results of the primary and secondary outcomes.

Outcomes	SALAD group (*n* = 18)	Intermittent suction group (*n* = 18)	Between-group difference	*p* value
CPR during the preintubation period				
Compression rate (bpm)^∗^	115.7 (113.8, 117.7)	110.9 (108.6, 113.3)	−4.8 (−7.8, −1.8)	< 0.01
Compression depth (cm)^∗^	5.0 (4.8, 5.2)	5.0 (4.8, 5.2)	0.0 (−0.3, 0.3)	0.94
Compression depth ≥ 5 cm	11 (61.1)	8 (44.4)		0.51
CPR during the intubation period				
Interruption time (seconds)^∗^	2.3 (2.1, 2.6)	2.5 (2.1, 2.9)	0.2 (−0.2, 0.6)	0.42
Compression rate (bpm)^∗^	112.9 (110.4, 115.4)	108.4 (105.7, 111.1)	−4.5 (−8.1, −0.9)	< 0.05
Compression depth (cm)^∗^	5.0 (4.7, 5.2)	4.9 (4.7, 5.0)	−0.1 (−0.4, 0.2)	0.52
Compression depth ≥ 5 cm	10 (55.6)	5 (27.8)		0.11
Intubation time (seconds)^∗^	16.1 (12.6, 19.6)	15.9 (12.2, 19.5)	−0.2 (−5.1, 4.6)	0.92
Intubation first-pass success	14 (77.8)	14 (77.8)		1.00
Esophageal intubation	1 (5.6)	4 (22.2)		0.34
The best glottic visualization before intubation				0.59
CL Grade 1	11 (61.1)	10 (55.6)		
CL Grade 2	7 (38.9)	7 (38.9)		
CL Grade 3	0 (0)	1 (5.6)		
CL Grade 4	0 (0)	0 (0)		
The degree of glottic obstruction during intubation				< 0.01
Grade 1 (0%)	13 (72.2)	4 (22.2)		
Grade 2 (0%–49%)	4 (22.2)	3 (16.7)		
Grade 3 (50%–99%)	0 (0)	8 (44.4)		
Grade 4 (100%)	1 (5.6)	3 (16.7)		

*Note:* CL, Cormack and Lehane classification. Data were presented as count (percentage) or mean (95% confidence interval)^∗^.

Abbreviations: bpm, beats per minute; CPR, cardiopulmonary resuscitation; SALAD, suction-assisted laryngoscopy airway decontamination.

**Table 3:** Within-group comparison of CPR quality before and during the intubation period.

Variables	Preintubation period	Intubation period	Within-group difference	*p* value
SALAD group				
Compression rate (bpm)	115.7 (113.8, 117.7)	112.9 (110.4, 115.4)	−2.8 (−4.4, −1.3)	< 0.01
Compression depth (cm)	5.0 (4.8, 5.2)	5.0 (4.7, 5.2)	−0.1 (−0.2, 0.0)	0.18
Intermittent suction group				
Compression rate (bpm)	110.9 (108.6, 113.3)	108.4 (105.7, 111.1)	−2.6 (−4.3, −0.8)	< 0.01
Compression depth (cm)	5.0 (4.8, 5.2)	4.9 (4.7, 5.0)	−0.2 (−0.3, −0.1)	< 0.01

*Note:* Data were presented as mean (95% confidence interval).

Abbreviations: bpm, beats per minute; CPR, cardiopulmonary resuscitation; SALAD, suction-assisted laryngoscopy airway decontamination.

## Data Availability

The data that support the findings of this study are available from the corresponding author upon reasonable request.

## Supporting Information

This video demonstrated the simulation process of the SALAD group.

## References

[1] Jost D., Minh P. D., Galinou N. (2015). What Is the Incidence of Regurgitation during an Out-Of-Hospital Cardiac Arrest? Observational Study. *Resuscitation*.

[2] Simons R. W., Rea T. D., Becker L. J., Eisenberg M. S. (2007). The Incidence and Significance of Emesis Associated with Out-Of-Hospital Cardiac Arrest. *Resuscitation*.

[3] Benger J. R., Kirby K., Black S. (2018). Effect of a Strategy of a Supraglottic Airway Device vs Tracheal Intubation during Out-Of-Hospital Cardiac Arrest on Functional Outcome: The AIRWAYS-2 Randomized Clinical Trial. *JAMA*.

[4] Wang H. E., Simeone S. J., Weaver M. D., Callaway C. W. (2009). Interruptions in Cardiopulmonary Resuscitation from Paramedic Endotracheal Intubation. *Annals of Emergency Medicine*.

[5] Ruetzler K., Gruber C., Nabecker S. (2011). Hands-off Time during Insertion of Six Airway Devices during Cardiopulmonary Resuscitation: a Randomised Manikin Trial. *Resuscitation*.

[6] Wang H. E., Jaureguibeitia X., Aramendi E. (2021). Airway Strategy and Chest Compression Quality in the Pragmatic Airway Resuscitation Trial. *Resuscitation*.

[7] Wang H. E., Schmicker R. H., Daya M. R. (2018). Effect of a Strategy of Initial Laryngeal Tube Insertion vs Endotracheal Intubation on 72-Hour Survival in Adults with Out-Of-Hospital Cardiac Arrest: A Randomized Clinical Trial. *JAMA*.

[8] Piegeler T., Roessler B., Goliasch G. (2016). Evaluation of Six Different Airway Devices Regarding Regurgitation and Pulmonary Aspiration during Cardio-Pulmonary Resuscitation (CPR)-A Human Cadaver Pilot Study. *Resuscitation*.

[9] Aldo F. L., Tighe D., Mort T. C. (2023). Airway Contamination: To Stop or Continue CPR during Intubation Attempts?. *Anesthesiology*.

[10] Lin L. W., DuCanto J., Hsu C. Y., Su Y. C., Huang C. C., Hung S. W. (2022). Compromised Cardiopulmonary Resuscitation Quality Due to Regurgitation during Endotracheal Intubation: a Randomised Crossover Manikin Simulation Study. *BMC Emergency Medicine*.

[11] DuCanto J., Serrano K. D., Thompson R. J. (2017). Novel Airway Training Tool that Simulates Vomiting: Suction-Assisted Laryngoscopy Assisted Decontamination (SALAD) System. *Western Journal of Emergency Medicine*.

[12] Lin L. W., Huang C. C., Ong J. R., Chong C. F., Wu N. Y., Hung S. W. (2019). The Suction-Assisted Laryngoscopy Assisted Decontamination Technique toward Successful Intubation during Massive Vomiting Simulation: A Pilot Before-After Study. *Medicine (Baltimore)*.

[13] Pilbery R., Teare M. D. (2019). Soiled Airway Tracheal Intubation and the Effectiveness of Decontamination by Paramedics (SATIATED): A Randomised Controlled Manikin Study. *British Paramedic Journal*.

[14] Ko S., Wong O. F., Wong C. H. K., Ma H. M., Lit C. H. A. (2021). A Pilot Study on Using Suction-Assisted Laryngoscopy Airway Decontamination Techniques to Assist Endotracheal Intubation by GlideScope® in a Manikin Simulating Massive Hematemesis. *Hong Kong Journal of Emergency Medicine*.

[15] Fiore M. P., Marmer S. L., Steuerwald M. T., Thompson R. J., Galgon R. E. (2019). Three Airway Management Techniques for Airway Decontamination in Massive Emesis: A Manikin Study. *Western Journal of Emergency Medicine*.

[16] Choi I., Choi Y. W., Han S. H., Lee J. H. (2020). Successful Endotracheal Intubation Using Suction-Assisted Laryngoscopy Assisted Decontamination Technique and a Head-Down Tilt Position during Massive Regurgitation. *Soonchunhyang Med Sci*.

[17] Frantz E., Sarani N., Pirotte A., Jackson B. S. (2021). Woman in Respiratory Distress. *JACEP Open*.

[18] Cormack R. S., Lehane J. (1983). Difficult Tracheal Intubation in Obstetrics. *Anaesthesia*.

[19] Kleinman M. E., Brennan E. E., Goldberger Z. D. (2015). Part 5: Adult Basic Life Support and Cardiopulmonary Resuscitation Quality: 2015 American Heart Association Guidelines Update for Cardiopulmonary Resuscitation and Emergency Cardiovascular Care. *Circulation*.

[20] Agostinucci J. M., Weisslinger L., Marzouk N. (2021). Relation between Chest Compression Rate and Depth: the ENFONCE Study. *European Journal of Emergency Medicine*.

[21] McAlister O., Harvey A., Currie H. (2023). Temporal Analysis of Continuous Chest Compression Rate and Depth Performed by Firefighters during Out of Hospital Cardiac Arrest. *Resuscitation*.

[22] Stiell I. G., Brown S. P., Nichol G. (2014). What Is the Optimal Chest Compression Depth during Out-Of-Hospital Cardiac Arrest Resuscitation of Adult Patients?. *Circulation*.

[23] Vittinghus S., Thomsen J. E., Harpsø M., Løfgren B. (2015). Does the Age of Medical Emergency Technicians Influence the Quality of Chest Compressions. *Scandinavian Journal of Trauma, Resuscitation and Emergency Medicine*.

[24] Voss S., Rhys M., Coates D. (2014). How Do Paramedics Manage the Airway during Out of Hospital Cardiac Arrest?. *Resuscitation*.

[25] Deakin C. D., Nolan J. P., Ji C. (2021). The Effect of Airway Management on CPR Quality in the PARAMEDIC2 Randomised Controlled Trial. *Resuscitation*.

[26] Donoghue A., Hsieh T. C., Nishisaki A., Myers S. (2016). Tracheal Intubation during Pediatric Cardiopulmonary Resuscitation: A Videography-Based Assessment in an Emergency Department Resuscitation Room. *Resuscitation*.

[27] Robinson A. E., Driver B. E., Prekker M. E. (2023). First Attempt Success with Continued versus Paused Chest Compressions during Cardiac Arrest in the Emergency Department. *Resuscitation*.

[28] Murphy D. L., Bulger N. E., Harrington B. M. (2021). Fewer Tracheal Intubation Attempts Are Associated with Improved Neurologically Intact Survival Following Out-Of-Hospital Cardiac Arrest. *Resuscitation*.

